# The Role of T Helper 22 Cells in Dermatological Disorders

**DOI:** 10.3389/fimmu.2022.911546

**Published:** 2022-07-14

**Authors:** Yu Pan, Dan Du, Lian Wang, Xiaoyun Wang, Gu He, Xian Jiang

**Affiliations:** ^1^ Department of Dermatology, West China Hospital, Sichuan University, Chengdu, China; ^2^ Department of Dermatology, the First Affiliated Hospital, Chongqing Medical University, Chongqing, China; ^3^ Laboratory of Dermatology, China Institute of Inflammation and Immunology (CIII), Frontiers Science Center for Disease-related Molecular Network, West China Hospital, Sichuan University, Chengdu, China; ^4^ State Key Laboratory of Biotherapy, West China Hospital, Sichuan University, Chengdu, China

**Keywords:** Th22 cells, IL-22, skin, inflammation, keratinocyte

## Abstract

T helper 22 (Th22) cells are a newly identified subset of CD4+ T cells that secrete the effector cytokine interleukin 22 (IL-22) upon specific antigen stimulation, barely with IFN-γ or IL-17. Increasing studies have demonstrated that Th22 cells and IL-22 play essential roles in skin barrier defense and skin disease pathogenesis since the IL-22 receptor is widely expressed in the skin, especially in keratinocytes. Herein, we reviewed the characterization, differentiation, and biological activities of Th22 cells and elucidated their roles in skin health and disease. We mainly focused on the intricate crosstalk between Th22 cells and keratinocytes and provided potential therapeutic strategies targeting the Th22/IL-22 signaling pathway.

## Introduction

The skin is the outmost sentinel barrier of the body and is continuously exposed to pathogens and the environment. The skin is composed of the epidermis, dermis, and subcutaneous tissues. Besides its mechanical barrier role, the skin acts as the first line of host immune defense. Keratinocytes are not only a component of the skin physical barrier but also have important immunoregulatory roles by secreting various cytokines and chemokines that work with cutaneous antigen-presenting cells (APC) such as Langerhans cells (LC), dendritic cells (DC), and macrophages, finally contributing to the innate immune system. To maintain skin immune homeostasis, innate and adaptive immune cells, especially T cells and tissue cells, interact finely with each other and constitute an intricate immune regulatory network (1, 2). As another leading actor of these crosstalk, dysregulated T cell immune responses can contribute to the pathogenesis of skin disorders. In the microenvironment and disease setting contexts, naive T cells can functionally differentiate into effector T helper (Th) cells. Furthermore, skin inflammatory diseases can be divided into four types based on the polarization of Th cells subsets. First, Th1-dominant skin diseases, such as lichen planus and vitiligo, share a lichenoid clinical phenotype. Atopic dermatitis (AD) is a clear example of Th2-specific skin diseases, characterized by autoantibody production and eosinophil-infiltrated eczematous patterns. Fibrogenesis is involved in regulatory T cells-polarized skin diseases, including systemic sclerosis. Finally, Th17 and Th22 cells, along with their cytokines IL-17 and IL-22, contribute to a neutrophil migration and wound-healing-like pattern, such as psoriasis (3–6).

The discovery of Th22 cells occurred much later than its defining cytokine, IL-22, which was initially regarded as a cytokine secreted by Th1 or Th17 cells. Nograles and Fujita proposed the possible existence of discrete Th22 cells, producing IL-22 without IFN-γ or IL-17 in the skin (7, 8). Then, Th22 cells were identified as a novel Th cell subset according to its particular transcriptional profile of aryl hydrocarbon receptor (AHR), chemokine receptors CCR4, CCR6, CCR10, as well as cytokines IL-22 instead of IFN-γ, IL-4, or IL-17 (9–11). Additionally, Th22 cells remain stable under inducible Th1, Th2, Th17, and Treg cells conditions, which reinforced that Th22 cells are a novel effector T subtype contributing to skin homeostasis and inflammation (10). Furthermore, results from an IL-17A fate-mapping reporter assay and a transcription profile supported Th22 cells as a distinct lineage from Th17 cells (12, 13).

Th22 cells have been proposed to play essential roles in autoimmune diseases including rheumatoid arthritis, inflammatory bowel diseases, asthma, multiple sclerosis, immune thrombocytopenia, nephropathy, hepatitis, thyroid diseases, and myasthenia gravis, as well as infectious diseases and tumors (14–22). Recently, emerging advances regarding the understanding of Th22 cells are allowing in-depth comprehension of the cutaneous immune system, which might promote the development of treatments targeting Th22 cells. Here, we review the current knowledge regarding Th22 cells and their main functional cytokine IL-22, especially their interaction with keratinocytes, in the pathogenesis of psoriasis, atopic dermatitis, lupus erythematosus, and other skin immune diseases. We also provided potential bases for treatments targeting the Th22/IL-22 signaling pathway.

## Characterization and Differentiation of Th22 Cells

Originally, Th22 cells were derived from the IL-22 study. IL-22 is the main effector cytokine of Th22 cells and belongs to the interleukin 10 (IL-10) family. IL-22 was first recognized as an IL-10-related T cell-derived inducible factor in 2000 (23, 24). Although IL-22 was primarily discovered that IL-22 was produced by Th1 and natural killer (NK) cells (25), IL-22 was usually viewed as a Th17 cytokine (26, 27) until the identification of Th22 cells (8–11). They were discovered as a new subset that only secreted IL-22, and barely produced IFN-γ, IL-4, or IL-17 (15, 20). Duhen estimated that about a half of IL-22 producing CD4+ memory T cells from healthy human peripheral blood secreted only IL-22, whereas around 30% and 13% separately co-expressed IFN-γ and IL-17, separately, with nearly 6% producing all three (9). Apart from its characteristic cytokine IL-22, Th22 cells can also produce IL-13 (10–12, 28), TNF-α (10, 11), IL-26 (11), and granzyme B (12). Additionally, Th22 cells orchestrate skin immunity and wound healing along with keratinocytes (10). Supporting the function of Th22 cells in the skin, they have been shown to possess skin-homing properties with CCR4, CCR6, and CCR10 expressions (9–11). Unsurprisingly, the frequency of cutaneous Th22 cells is higher in inflammatory skin than that in the blood, accounting for 30% of IL-22+ cells in the epidermis and dermis (10). Recently, Foster’s team generated IL17aeGFPIL22tdTom dual-reporter transgenic mice and proved that Th22 cells were not derived from Th17 cells as a distinct lineage in Th22-enriched culture conditions (12). Further, they validated this finding *in vivo* with triple-reporter mice in which Th22 cells seldom developed through the IL-17A-expressing stage during bacterial infection (13). Collectively, Th22 cells are regarded as a novel T cell subset. Considering the complexity of T cells subsets identification, flow cytometry appeared to be the most popular method to segregate Th22 cells by combining extracellular proteins (CCR4, CCR6, and CCR10), a transcription factor (AHR) and cytokine profiles (IL-22+, along with IFN-γ-, IL-4-, IL-9-, IL-10- and IL-17-) (29). Additionally, the dual cytokine-secretion assay allowes the isolation and purification of Th22 cells, which might provide possibilities for further characterizations (30).

**Figure 1:**
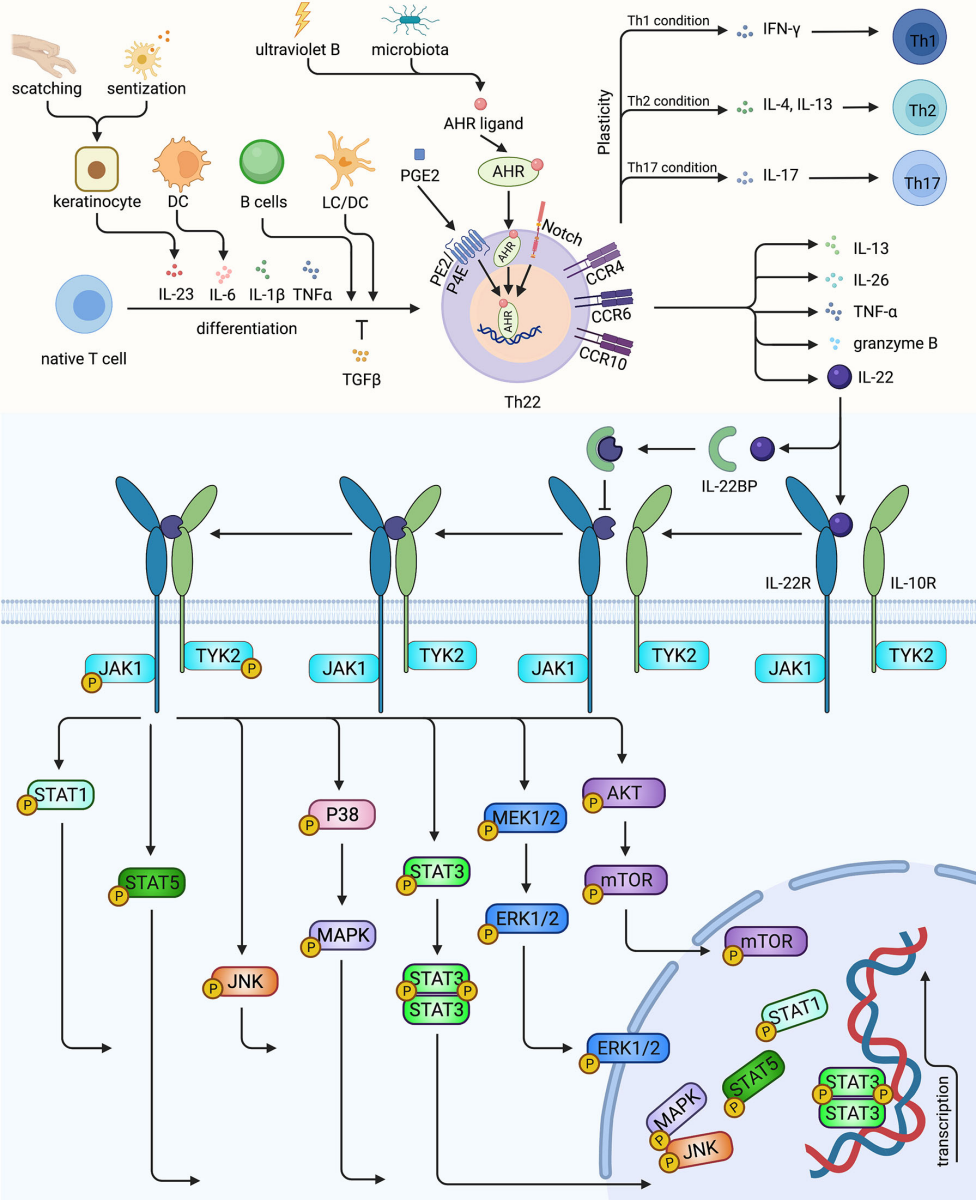
The characterization, differentiation of Th22 and the signaling pathway of Th22/IL-22. Native T cells induced by IL-6, IL-23, IL-1β and TNF-α, mediated by LC, DC or activated B cells could differentiate into Th22, while TGFβ could inhibit Th22 polarization. AHR, the master transcription factor of Th22, could be upregulated by ultraviolet B, microbiota, PGE2 and Notch signaling. Th22 secreted its effector cytokine IL-22, as well as IL-13, IL-26, TNF- α and granzyme B. Th22 might converted to Th1, Th2, Th17 under certain conditions. IL-22 could bind to receptor complex of IL-22R and IL-10R, activating TYK2 and JAK1, thus mediating multiple pathways. In contrast, the decoy receptor IL-22BP blocks the roles of IL-22. Th22, T helper 22 cell; LC, Langerhans cell; DC, dendritic cell; AHR, aryl hydrocarbon receptor; IL-22R, IL-22 receptor; IL-10R, IL-10 receptor; IL-22BP, IL-22 binding protein; PGE2, Prostaglandin E2. Created with Biorender.com.

The differentiation of T cells is primed by direct contact or the interaction with cytokines produced by APC and tissue cells and depends on complex lineage-defining transcriptional networks (31). Previous studies have demonstrated that TGF-β promotes IL-17 while differentially inhibits IL-22 during early Th cell differentiation (11, 32). Trifari has also found that human naive T cells co-cultured with IL-1β and IL-23 secreted IL-22 rather than IL-17 (11). Further, Yoon has revealed that tape stripping of mouse and human skin can lead to the activation of Toll-like receptors 4 (TLR4). Finally, the skin DC can be polarized by IL-23 and promote Th22 cell differentiation (33). On the other hand, Basu has shown that IL-6 alone can trigger the development of Th22 cells. (34). Although IL-23 can augment the polarization of Th22 cells primed by IL-6, IL-23 is dispensable since its blockage did not affect TCDD-induced IL-22 production. (34, 35). Moreover, Duhen has demonstrated that IL-6 and TNF-α contributed to Th22 cells production (9). Studies also suggested that IL21 or IL-26 enhanced Th22 differentiation (36, 37). Recently, Prostaglandin E2 (PGE2) has been proved to increase IL-22 under Th22-priming conditions and to stimulate IL-22 *in vivo* by binding to the EP4 receptor. (38). Prostaglandin I (2) analogs has been proved to augment IL-22 and IL-17 *in vitro* and expanded Th17 and TH22 cells as well (39). Jiao et al. have found that ICOS/ICOSL signaling increased the proliferation of CCR4+ CCR6+ Th22 cells (40). Besides various cytokines and molecules, APC could also regulate Th22 cells differentiation (**Figure 1**). Plasmacytoid DC (pDC) was more potent than conventional DC (cDC) to induce human Th22 cells *via* IL-6 secretion (9). For example, Wilkinson has shown that IL-6 is released by DC-polarized Th22 cells through classical, trans, and cluster signaling pathways. (41). Additionally, CD5+ LC and CD5+ DC were strong stimulators of Th22 cells (42). In another study, LC was more efficient than DC to induce Th22 polarization (8). Yang indicated that activated B cells also promoted Th22 cell formation (43). Although studies revealed that various factors regulated Th22 cells differentiation, there was no standard Th22 cells polarization protocol. Recently, the optimized Th22-skewing condition proposed by Plank was widely accepted, which was composed of IL-23, IL-1β, IL-6, the endogenous AHR ligand 6-Formylindolo[3,2-b]carbazole (FICZ), and the TGF-βR inhibitor Galunsertib (12). However, differentiation of Th22 cells in different context of disease settings and distinct immune microenvironments remains to be explored.

The transcriptional mechanisms orchestrating the phenotypic identity of Th22 cells are not fully elucidated. Nevertheless, AHR is mainly considered as a characteristic transcription factor of Th22 cells. Studies have demonstrated that the AHR agonists β-naphthoflavone, FICZ, and dioxin upregulated not only induced the production of IL-22 but also of IL-17 (11, 35). Similarly, the AHR ligand VAF347 selectively promoted IL-22 by acting on monocytes and naive T cells (44). Conversely, inhibiting or silencing AHR *in vitro*, and knocking out AHR *in vivo* led to reduced IL-22 (11, 34, 45). Moreover, Alam et al. have identified a Notch-AHR axis using RBP-J deficient mice, where Notch1 and Notch2 compensated each other to control IL-22 expression. They have also discovered that Notch signaling can induce heat-labile molecules to stimulate AHR (45). Zeng has also shown that the Notch-Hes-1 axis facilitates the production of Th22 cells (46). Medroxyprogesterone acetate promoted Th22 cells through AHR and T-bet while inhibiting RORγt (47). Besides the essential roles of master regulatory, co-regulator, placeholder, and other epigenetic programs also cooperate together in a coordinated network to drive Th cells differentiation. For example, RORγt is a positive regulator of Th22 cell differentiation, (11, 12). combined with RUNX1, it promotes IL-22 expression through their enhancer, CNS-32 (48). Besides, HIF-1α and RUNX3 can also elevate the expression of IL-22 (49, 50). Notably, T-bet knock-out mice exhibited compromised IL-22 expression in Th22 cells without affecting IL-22 in Th17 cells (34). In contrast, Plank has described the inhibitory role of T-bet *in vitro* in Th22 cell cultures with potential minimal Th17 cell contamination (12). These controversial results motivated us to define Th22 cells accurately and explore the underlying mechanisms balancing their stability and plasticity.

Plasticity allows for heterogeneity, which facilitates Th cells to continuously adapt to the ever-changing surroundings and is crucial for immune homeostasis (31, 51). Studies have demonstrated that a proportion of Th22 cells can express IFN-γ (10, 12, 28, 32), IL-4 (10) IL-13 (12, 28), and IL-17 (10, 12, 13, 32), indicating a possible conversion from Th22 cells to Th1/2/17 cells. In turn, Th17 clones, derived from psoriatic skin, could be polarized to an IL-22-single-producing Th22 cell profile (52). Besides, T cells exposed to filaggrin-deficient skin equivalents shifted from a Th1/Th17 to a Th2/Th22 profile (53). Additionally, IL-22+ Th cells predominantly produce IFN-γ instead of IL-17 under Th0 and Th17 conditions, indicating co-regulation of IL-22 and IFN-γ (32). Shen et al. have described that *in vitro* generated mouse IL-22+ Th cells were relatively stable under Th1 or Th2 conditions *in vitro*, whilst acquired IL-17-expressing ability under Th17 conditions (54). However, Plank et al. have suggested that IL-17, IL-13, and IFN-γ expressions were augmented in Th22 cells under respective re-stimulated circumstances (12). The different polarization conditions that Shen used to differentiate Th22 cells with TGF-β while Plank induced Th22 differentiation with TGF-βR inhibitor Galunisertib with potential minimal Th17 contamination, which might account for the paradoxical results. The *in vivo* generated IL-22+ Th cells in IL-22-tdTomato mice lost IL-22 expression and converted to express IL-17 with higher T-bet. This was further confirmed by a colitis model in which transferred IL-22+T cells gained IFN-γ and IL-17 productions (54). Furthermore, IL-22+ Th cells generated *in vivo* using IL-22-tdTomato mice lost IL-22 expression and expressed IL-17 with higher T-bet, which was further confirmed by a colitis model in which the transferred IL-22+T cells started to produce IFN-γ and IL-17 (12). Human study proved that the plasticity of Th22 cells varied in different diseases with dysregulated Th-skewing. IL-4-secreting Th22 cells were only apparent in atopic eczema rather than allergic contact dermatitis or psoriasis, although IFN-γ and IL-17-producing Th22 cells could be found in all three (10). In summary, Th22 cells can acquire plasticity under certain influences including extrinsic factors like cytokine milieu or pathogen infection, which might play essential roles in the pathogenesis of skin immune diseases. Finally, uncovering the mechanisms that orchestrate the differentiation and plasticity of Th22 cells is crucial for therapeutic interventions.

## Biological Activities of Th22 Cells and IL-22 in the Skin

As mentioned above, Th22 cells were initially discovered and play a vital role in the skin, particularly *via* its effector cytokine, IL-22. In addition to Th22 cells, cutaneous IL-22 is also expressed by innate and adaptive immune cells including NK cells, innate lymphoid cells (ILCs), lymphoid tissue inducer cells, NK T and γδT cells, DC, macrophages, mast cells, neutrophils, as well as Th1 cells, Th17 cells, and CD8+ T cells, and even fibroblasts (55–63) (**Table S1**). Generally, innate immune cells respond directly to local environment and quickly modulate surrounding epithelial host defense in the early state, while T cells induce strong and sustained immune responders in the late state. ILCs were supposed as the main producer of IL-22 in mucosa, while Th cells were believed as the primary sources of IL-22 in epithelial tissues (64). ILCs initiated inflammatory response rapidly after stimulation by IL-23 in psoriatic mice (65). Compared with ILCs targeting surface intestinal epithelial cells, Th22 cells have a nonredundant role in the intestinal crypts, revealing spatiotemporal differences between Th22 cells and ILC3 during infection (66). The differences of IL-22-producing ILCs and Th22 cells were not fully elucidated in the skin. ILCs were mostly clustered beneath the dermoepidermal junction and close to T cells (67), which indicated possible crosstalk between them. Strikingly, Mashiko proposed that mast cells were major IL-22 producers in psoriasis patients and atopic dermatitis individuals (68). The emergences of IL-22-expressing cancer-associated fibroblasts and immunofibroblats enhance the complexity of IL-22 production. The limited data requires further exploration of the different roles of various IL-22-expressing cells, both in physiological and pathological conditions. Questions such as whether IL-22-expressing innate immune cells respond earlier and result in acute inflammation while IL-22-producing T cells participate in later stages and cause chronic immune dysfunction, and what are the differences between Th17/Tc17 and Th22/Tc22, considering differentiation, regulation, and plasticity in specific pathological conditions remains unanswered. Also, it is important to consider the differences between murine and human skin when interpreting the underlying mechanism of skin disorders. For example, it is known that the interfollicular epidermis in humans is larger than that in mice. Moreover, the composition of their immune cells is different. For example, dendritic epidermal T cells are only present in the mouse skin. Here, we summarized published data and focused on the biological activities of Th22 cells and IL-22 in the skin.

**Figure 2:**
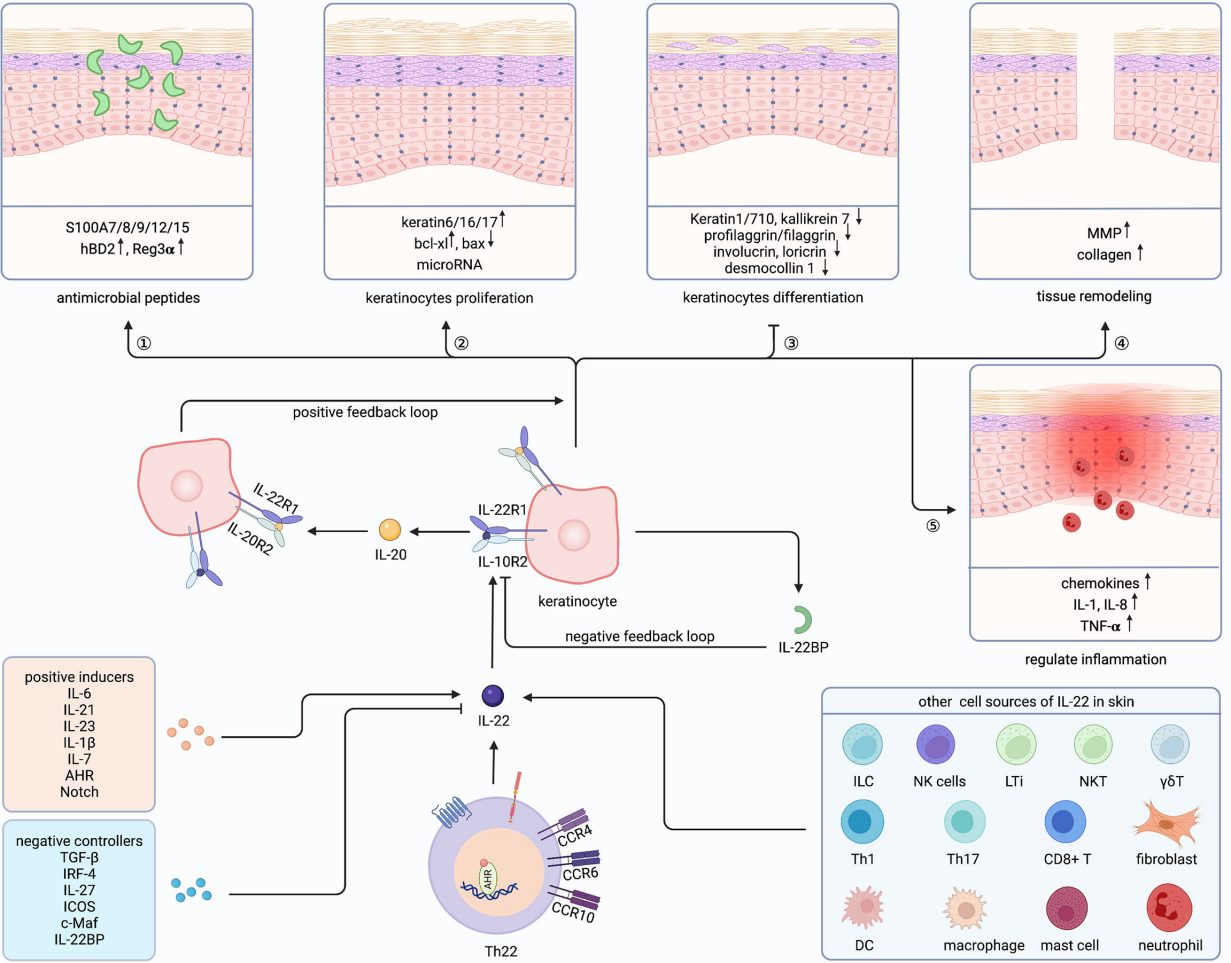
Cutaneous biological roles of Th22 cells and IL-22. Th22 cells exert biological functions through its effector cytokine IL-22 in the skin. Besides, IL-22 could also be secreted by ILC, NK cells, LTi, NKT, γδT, Th1/17, CD8+ T cell, DC, macrophage, mast cell, neutrophil and fibroblast in the skin. Both positive inducers and negative controllers could regulate the expression of IL-22. IL-22 plays a vital role in Th22-mediated host defense by communicating with keratinocytes, promoting antimicrobial peptides and keratinocytes proliferation, inhibiting keratinocyte differentiation, and regulating tissue remodeling as well as inflammation. Furthermore, IL-22 could amplify its biological activities through a positive feedback loop by inducing IL-20 production in keratinocytes, which has similar effects on keratinocytes. ILC, innate lymphoid cell; NKT, natural killer T cell; LTi, Lymphoid tissue inducer; γδT, gamma-delta T cell; DC, dendritic cell; Th, T helper cell; IL-22BP, IL-22 binding protein; MMP, matrix metalloproteinases; AHR, aryl hydrocarbon receptor. Created with Biorender.com.

IL-22 can be regulated by positive inducers including IL-6, IL-21, IL-23, IL-1β, IL-7, AHR, Notch and negative controllers such as TGF-β, IRF-4, IL-27, ICOS, and c-Maf (**Figure 2**). Interestingly, ultraviolet B irradiation of keratinocytes increased the productions of both AHR ligand (69, 70) and IL-22R1 (71), amplifying the roles of Th22 cells and IL-22 (**Figure 1**). Soluble IL-22 binding proteins (IL-22BP, or IL-22R2) serve as its natural inhibitor. Furthermore, a high concentration of IL-38 augmented IL-22 while a low concentration attenuated it (58, 60, 63, 72). IL-22 binds with a heterodimeric complex receptor composed of IL-22R1 and IL-10R2. Previously, it was determined that IL-22 bound to IL-22R1 first with high affinity, leading to a conformational adjustment which in turn promotes the combination with IL-10R. While IL-10R2 is widely expressed, IL-22R1 is restricted to non-hematopoietic epithelial cells such as keratinocytes and fibroblasts. IL-22 activates JAK1 and TYK2, transmitting the signal to downstream pathways, including STAT3, STAT1, STAT5, P38, MAPK, JNK and MEK, ERK, AKT, and mTOR (58–60, 63, 72, 73) (**Figure 1**). Considering the expressions of IL-22R1 and IL-22BP in keratinocytes, IL-22 functions to sustain the integrity and barrier functions of the skin. In contrast, IL-22 can also amplify inflammation cascade, and promote abnormal proliferation and differentiation in certain cases.

IL-22 plays a vital role in Th22-mediated host defense by communicating with keratinocytes (**Figure 2**). The function of IL-22 on keratinocytes can be divided into five sections. First, IL-22 protects the host defense through promoting antimicrobial peptides (AMP), including psoriasin (S100A7), calgranulin A (S100A8), calgranulin B (S100A9), S100A12, S100A15, hBD2, and hBD3 (26, 74–77). Similarly, keratinocytes exposed to Th22 supernatants showed increased S100A7 (10). Mechanically, MSX2P1 promoted the progression and growth of IL-22-stimulated keratinocytes by targeting miR-6731-5p and activating S100A7 (78). Additionally, IL-22-induced SPRR2C has been reported to target the miR-330/STAT1/S100A7 axis in HaCaT cells (79). In contrast, CXCL8 and hBD2 were upregulated by IL-22 *via* STAT3-mediated Bcl-3 (77). Interestingly, IL-17 alone could not enhance S100A7 and S100A8, but, when combined with IL-22, upregulated S100A9 and hBD in primary keratinocytes (26). Unlike the described host defense role of IL-22 to induce Reg family proteins in intestinal epithelia, IL-22 alone could not induce Reg3α in keratinocytes (80). However, along with IL-17, IL-22 can elevate the levels of Reg3α, thereby promoting the proliferation of keratinocytes and inhibiting the terminal differentiation in feedback (81). Blocking either IL-17 or IL-22 increased the severity of Staphylococcus aureus infections, indicating complementary and nonredundant roles of IL-17 and IL-22 (82).

Second, IL-22 promotes keratinocytes proliferation. Among the IL-20 family, IL-22 induced the maximal level of proliferation both in monolayer keratinocytes and reconstituted human epithelial culture system (76). Yang proved that Nrf2 elevated keratin 6 (K6), K16, and K17 by regulating their antioxidant responsive element after IL-22 stimulation, finally promoting proliferation (83). By contrast, IL-22 inhibited apoptosis by increasing Bcl-xl and decreasing Bax (84). Recently, studies demonstrated that microRNAs (miRNAs) cooperated with IL-22 and regulated keratinocytes proliferation through miR-197/IL-22R, miR-122-5p/Sprouty2, miR-330/CTNNB1, miR-20a-3p/SFMBT1, miR-548a-3p/PPP3R1, MSX2P1/miR-6731-5p, miR-233/PTEN, NORAD/miR-26a, and miR-617/FOXO4 signaling pathways (78, 85–92). Furthermore, STAT3, Erk, Akt, MAPK, and JNK were demonstrated to be involved in the hyperproliferation of keratinocytes (93–97). Notably, IL-22 and IL-17 might induce keratinocytes stemness to promote regeneration (98).

Third, IL-22 can restrain the differentiation of keratinocytes and has essential impacts on epithelial architecture, leading to acanthosis, parakeratosis, and hypogranularity. Those differentiated proteins were inhibited by IL-22, including K1, K10, K7, kallikrein 7, profilaggrin/filaggrin, involucrin, loricrin, desmocollin 1, and CALM5 (75, 76, 99–103). Padhi further proved that IL-22 suppressed peptidylarginine deiminase-1 to hinder epithelial differentiation (104).

Fourth, IL-22 also contributes to tissue remodeling, particularly through matrix metalloproteinases (MMP) 1 and 3 in keratinocytes (75, 99, 105). Scratch wound healing and transwell assay further proved the mobility role of IL-22 (95, 103). Impaired skin showed a delayed healing process and reduced IL-22 (105, 106), while recombinant IL-22 alleviated skin damage in different wound-healing stages (103, 107). Additionally, IL-22 promoted collagen deposition in fibroblasts and accelerated wound healing (73, 108). Moreover, IL-22 activated TNF-induced keratinocytes, then promoted MMP1 in fibroblasts through increasing IL-22R (109). Recently, Li suggested that CD11c+ myeloid cells were important for the underlying mechanisms of wound healing *via* regulation of the IL-23/IL-22 axis (110).

Fifth, IL-22 can also regulates inflammation in keratinocytes, mainly through inducing granulocyte-attracting chemokines, such as CXCL1, CXCL5, and CXCL8 (76, 99, 111). Also, IL-22 can enhance IL-1, IL-8, and TNF-α (71, 112–114). Furthermore, IFNα increased IL-22R and amplified the pro-inflammatory role of IL-22 (112, 115). Interestingly, IL-22, IL-20, and IL-24 share the IL-22R1 subunit and have redundant roles in the skin (76, 100, 107). Moreover, IFNα increases IL-22R and amplifies the pro-inflammatory role of IL-22 (100).

Nowadays, increasing studies have proved that IL-22 exerted dual roles, both guardian and pathogenic, depending on the context (56, 63, 116). The dysregulated interplay between immune and epithelial cells can lead to the disruption of skin homeostasis. In contrast to its skin integrity protective function, excessive IL-22 can lead to skin disorders. There is emerging evidence that Th22 cells and IL-22 are involved in the development of skin diseases, particularly psoriasis and AD (55, 57, 62).

IL-22 is thought to multifactorial develop psoriatic lesions. Also, the pathological pattern of psoriasis can be viewed as an uncontrolled IL-22 physiological activity in all aspects. Gene-editing mice are widely used when exploring the mechanism of psoriasis-like models composed of hyperkeratosis, parakeratosis, acanthosis, and dermal inflammation. IL-22-transgenic mice showed neonatal mortality and psoriatic morphological changes (117). Reversely, IL-22-deficient mice were protected from IL-23- or imiquimod-induced psoriatic lesions (27, 118, 119). Similarly, neutralizing IL-22 significantly ameliorates murine psoriatic phenotype (27, 119, 120). Moreover, knocking down IL-22BP also elevates the production of Th22 and γδT cells, (121, 122). thereby deteriorating psoriasiform dermatitis. (121, 123). Zheng suggested that IL-23 induced IL-22 production and activated STAT3, while IL-6 was dispensable for IL-22 expression (27). Then, Lindroos verified that the expressions of IL-22R1 and IL-22BP were IL-6-dependent, although IL-6-/- T cells did not reduce IL-22 production (124). On the other hand, Chen has shown that K23/IL-22^+/+^ and K23/IL-22^−/−^ mice are not different and hypothesized that IL-22 deficiency is not required for psoriasis development, although IL-22 aggravates psoriatic arthritis in K23 mice. (125). Hedrick demonstrated that IL-23 did not induce IL-22 in CCR6-/- mouse ears, while triggering IL-22 production in Rag-/- mice, implying that both T and non-T cell-derived IL-22 were involved in IL-23-induced ear swelling in murine psoriasis models (126). The previous studies were predominantly concentrated on the biological activities of IL-22 and simply considered it as a Th17 cytokine in the IL-23/IL-17/IL-22 axis in psoriasiform models without elucidating the sources of IL-22. Along with IL-22, IL-17, the primary effector of Th17 cells, also seemed necessary to induce IL-23-mediated murine psoriasis (118, 127). A 2D visualization of psoriasis RNA-seq datasets was unable to prove the relationship between IL-17A and IL-22, which indicated alternative sources of IL-22 such as Th22 or non-T cells rather than Th17 in psoriasis (128). Th22 and IL-22 producing CD8+ T cells are one of the primary sources of IL-22 in AD (129). For example, IL-22 mediates the proliferation of keratinocytes and epithelial thickness in AD-like models. (130). IL-22 mediated keratinocytes proliferation and epithelial thickness in AD-like models (131–133). Additionally, skin-specific IL-22 transgenic (K5-tTA-IL-22) mice exhibited chronic pruritic dermatitis with increased IL-4, IL-13, and gastrin-releasing peptides, along with elevated susceptibility to S. aureus infections (134). Epicutaneous-sensitized AD models promoted IL-22 expression as well as Th22 cell accumulation (33, 135–137). IL-23, triggered by endogenous TLR4, promoted DC-induced Th22 cell polarization after antigen application following tape stripping in mouse skin (33). Furthermore, Robb found that PGE2 markedly augmented IL-22 in both Th22 cells and ILCs (38, 138). Several studies proposed that ILCs and γδT cells were acknowledged as primary producers of IL-22 in psoriatic mouse models instead of Th cells (65, 119, 139–141).

## The Roles of Th22 Cells in Skin Diseases

Studies have revealed that Th22 cells play a regulatory role in the pathogenesis of varied immune disorders, infections, and tumors (14, 15, 21, 22, 142, 143). Here, we provided a brief summary of published research pertaining to the roles of Th22 cells in skin diseases.

### Psoriasis

Psoriasis is an inflammatory skin disease with high incidence, characterized by erythematous scaly patches or plaques usually on extensor locations, caused by immune-mediated hyperproliferation and chaotic differentiation of keratinocytes. Substantial progress has been made in relating skin lesions with systemic comorbidities such as psoriatic arthritis (PsA) and metabolic syndromes (144). Both circulation and skin lesion showed higher IL-22 expression in psoriasis patients, which was also associated with disease severity (84, 145–148). Although IL-22-producing Th22 cells were first discovered in psoriatic lesions, it is widely accepted that IL-17- and IL-22- expressing Th17 cells drive the inflammatory cascades and activate and recruit Th1 cells and Th22 cells, resulting in abnormal immune response and aberrant epidermal proliferation in psoriasis (149). Moreover, IL-22 enhanced the production of IL-17 in keratinocytes, promoting keratinocytes migration (150). IL-17 and IL-22 in synergism with each other induce the secretion of AMP, promote acanthosis, parakeratosis and inflammation. However, while IL-17 dominates inflammation by activating immune cells, IL-22 mainly regulates the proliferation and differentiation of keratinocytes.

IL-22 might be a genetic risk factor in psoriasis. Copy number variations (CNV) of IL-22 exon1 were significantly associated with psoriasis severity (151, 152). Pollock proposed IL-22 as a possible germ line risk locus for PsA (153) Furthermore, Nikamo found that a high-risk IL-22 promoter variant might lead to the onset of psoriasis before puberty (154). Similarly, Cordero discovered higher IL-22-expressing T cells in pediatric psoriasis patients rather than adult ones (155). Both studies indicated that pediatric psoriasis patients might benefit from anti-IL-22 therapy. Besides, differential IL-22 expression at different sites might result in diverse local manifestations of psoriasis. Serum IL-22 was elevated in PsA patients with entheseal and joint changes (148, 156). CD4+ IL-22+ T cells were lower in the synovial fluid of PsA (156), while the level of IL-22 was higher in the intestine of PsA patients (157). Moreover, Ahn found that scalp psoriasis had higher IL-22 derived from CD8+T cells (158). However, using GSEA and GSVA, Ruano has found a similar Th1/Th17 profile but less activation of Th22 cells in scalp psoriasis compared to skin psoriasis (159). Besides, these discordant expressions at different sites put forward a hypothesis that differential IL-22 expression might result in diverse local manifestations of psoriasis.

Collectively, both IL-22-producing Th17 cells and Th22 cells play important roles in psoriasis patients. Rather than dominant function of Th17 cells in psoriasis, Th22 cells, serve as immune response of Th17 cells, mostly act on keratinocytes leading to excessive proliferation and deviate differentiation in the skin. However, the emergence of IL-22-producing innate immune cells increase the complexity of immune activity in psoriasis. Future studies illustrating distinct roles among innate and adaptive IL-22-expressing immune cells are required.

### AD

AD is the most common pruritic inflammatory skin disease with recurrent eczematous dermatitis. AD affects all ages and ethnicities and can be classified as extrinsic and intrinsic phenotypes. Its pathophysiology is complicated and involves genetic predisposition and epidermal dysfunction, along with diverse T cell-related inflammation (160).

Numerous Studies demonstrated that increased expression of IL-22 was correlated with the Scoring of Atopic Dermatitis (SCORAD) (161–164). The elevated IL-22 was localized in skin lesions in mild AD (165), while affecting both local skin and circulation in severe AD (162). A comparative study revealed differential expression of chemokines in AD (CCL17/18/20) and psoriasis (CXCL1, IL-8, and CCL20), indicating that disease-specific microenvironments might be involved in Th differentiation (166) While psoriasis is Th17/Th22-skewing, AD tends to be Th2/Th22 polarized (129, 166, 167). The Th2-skewing microenvironment inhibited AMP, thus resulting in more vulnerable skin colonization with *S. aureus* in AD patients rather than in psoriasis individuals (162, 168).

The immune infiltration in AD is quite heterogeneous depending on the disease-specific manifestation-related contexts. Intrinsic AD presented higher skewing of Th17/TH22 than extrinsic phenotype (169), and the “Th2/Th22/PARC-dominant” cluster in four types of intrinsic AD patients showed the highest SCORAD (170). Besides, Th2/Th22 activation progressively intensified from acute to chronic while IL-17 was decreased from acute to chronic phase (168, 171). The immune cells infiltrations also vary among different AD ethnicities. African American AD patients (172), Tanzanian AD patients (173), and Asian AD patients (174, 175) had higher Th2/Th22 skewing than European Americans. Although common Th2/Th22 skewing can be found in different age groups (176), adult AD patients had elevated IL-22 frequencies than infants and adolescents (177, 178) but decreased with age in elderly AD patients especially those over 40 (179, 180). Future study with continuous monitoring in AD patients from infants to seniors might expand our knowledge in aged-related immune heterogeneity in AD. Moreover, gene predisposition influences the immune pathways in AD. AD children with FLG mutation demonstrated higher Th22 cell numbers without affecting the adaptive immunity composition (181).

Recently, laser capture micro-dissection has been used to detect IL-22 in the dermis (163). Compared to a skin biopsy, tape strip sampling and interstitial fluid from suction blistering are less invasive methods to identify similar inflammatory cytokines (182–184). However, the detection and qualification of cytokine profiles in interstitial fluid is unstable and sometimes might not detect IL-22 (185). Microneedle patch for ultrasensitive biomarkers quantification in interstitial fluids would facilitate point-of-care diagnosis and longitudinal monitoring and uncover the underlying mechanism of background immune activation in different ethnicities and age groups.

### Lupus Erythematosus

Lupus erythematosus is a heterogeneous autoimmune disease, ranging from skin lesions to nephritis, characterized by exaggerated B and T cell immune responses with impaired immune tolerance against self-antigens (186–188). Studies related with Th22 cells and IL-22 in lupus patients are quite controversial, possibly due to small sample sizes.

Some found decreased plasma IL-22 in inactive SLE individuals (189) and new-onset SLE patients (190). In contrast, other studies revealed elevated Th22/IL-22, implying its pathologic roles (191, 192). CNV of IL-22 and IL-17 were higher in SLE patients without synergistic contribution to SLE risks (193). Additionally, IL-22 gene rs2227513 polymorphism was associated with increased risk in renal SLE (194). The frequencies of Th22 cells vary among different lupus erythematosus subtypes, indicating a prognostic role of IL-22 in various types. Additionally, discoid erythematosus has higher levels of Th22 cells and IL-22 compared to subacute cutaneous lupus erythematosus in both skin lesions and circulation. (195, 196). Yang et al. have also found increased Th22 cells in sole skin involvement patients but decreased in sole nephritis ones (197). However, they later reported controversial results in which serum IL-22 levels in AD were higher compared to healthy controls, and anti-IL-22 monoclonal antibody treatment attenuated nephritis in MRL-lpr mice (198). Zhong found augmented CCR6+ Th22 cells in SLE patients with sole skin and/or renal impairments (199). In addition, Luk investigated and revealed higher IL-22 levels in the urinary sediment of SLE patients with proliferative nephritis than those with nonproliferative (200).

### Other Skin Inflammatory Immune Diseases

Systemic sclerosis (SSc) is an immune-mediated disease with T cell abnormalities, characterized by skin or/and organs fibrosis. SSc patients had increased Th22 cells in both circulation and lesional skin, indicating abnormal crosstalk between Th22 cells and fibroblasts (39, 108, 109, 201, 202). Hypertrophic scar and keloid caused by proliferative fibroblasts further confirmed the interaction between Th22 cells and fibroblasts with augmented Th22 numbers and IL-22 levels (195, 203, 204). Alopecia areata (AA) is a common non-scarring hair-loss immune disease with limited therapeutics (205). Some studies described elevated IL-22 in both AA lesions and serum (206), as well as expanded CLA+ Th22 cells in the blood (207). Meanwhile, other studies have suggested AA as Th1/Th2 skewing instead of Th17/Th22 (208). Larger cohort investigations are urgent to reveal underlying Th polarization in AA. The biology of Behcet’s disease (BD) is not fully understood, however, a recent study found that increased Th22 cells were associated with mucocutaneous BD (209). Hidradenitis suppurativa (HS) is a chronic inflammatory disorder with painful inflamed nodules, abscesses, sinus tracts, and fistulas in intertriginous skin, leading to severely decreased life quality (210). IL-22 deficiencies might participate in HS pathogenetic mechanisms (211, 212). In contrast, transcriptional profiles showed apparent Th22/IL-22-skewing in prurigo nodularis (213). Furthermore, Th22 cells were augmented in Graft-versus-host disease (GVHD), a complication following allogeneic hematopoietic-cell transplantation involving the skin (214, 215). IL-22 derived from donor T- cells alleviated cutaneous GVHD in mouse models (216, 217).

Taken together, Th22 cells and IL-22 play essential roles in in the pathogenesis of skin disorders. However, we found that most studies and clinical trials (**Table S2**) concentrate on the function of IL-22. As we know, IL-22 could be secreted by not only Th22 cells but other immune cells and tissue cells. Thus, future studies focused on the behaviors of Th22 cells n dermatological disorders are needed.

## Biologics Targeting IL-22 Signaling Pathways in Skin Diseases

Over the past 20 years, biologics have remarkably updated our knowledge of dermatological treatments, especially in psoriasis and AD. Biologics are recommended as a first-line treatment of moderate to severe plaque psoriasis, including 11 approved inhibitors of TNF-α (infliximab, adalimumab, certolizumab pegol, golimumab, and etanercept), p19 of IL-23 (tildrakizumab, risankizumab, and guselkumab), p40 of IL-12/23 (ustekinumab), IL-17(secukinumab, ixekizumab, and brodalumab) (144, 218, 219). Another two drugs targeted IL-23 or IL-17, Mirikizumab (anti-p19 of IL-23) (220) or bimekizumab (anti-IL-17) (221), are in clinical development. Currently, three inhibitors are approved in AD, including dupilumab (anti-IL-4Rα), tralokinumab (anti-IL-13), baricitinib (JAK1/2 inhibitor). Antibodies of CCR4, CCL20, IL-6/IL-6R, along withother JAK inhibitors (detailed information see **Table S2**) are under clinical development. However, all of those biologics are not applicable in patients with active tuberculosis or hepatitis B virus infection, which might lead to deadly infection (218, 222, 223). Targeting the IL-22-IL-22R system might provide a new therapeutic with minimal side effects of infection due to the lack of IL-22R on hematopoietic cells (59).

Reich found that guselkumab had better long-term efficacy than secukinumab (224), which might because of broader spectrum of both IL-22 and IL-17 inhibiting roles of guselkumab than decrising Il-17 solely of sekinumab. Furthermore, Evidences of suppression of IL-22 expression were also discovered in infliximab (225), adalimumab (225–227), etanercept (228, 229), tildrakizumab (230), risankizumab (230), guselkumab (231, 232), ustekinumab (233–235), filgotinib (jak1 inhibitor) (236), upadacitinib (jak1 inhibitor) (230) and baricitinib (237), which is in consistent with the signaling pathway of Th22/IL-22. Besides, ruxolitinib exhibited extra role of inhibiting expression of IL-22R1 (238) (**Table S3**). In contrast, anakinra (recombinant IL-1Rα) increased production of IL-22 in Hidradenitis Suppurativa (239, 240), possibly *via* promoting the differentiation of Th22 cells. However, tapinarof (AHR agonist) increased the production of IL-22 in keratinocytes *in vitro*, while inhibited IL-22 in psoriasis mouse model (241). This discrepancy again reminds us of the complexity of IL-22 related immune responses with both innate and adaptive IL-22-producing immune cells, which urges us to clarify the different roles of IL-22-producing cells in human health and skin diseases.

Integrative biology techniques combing human transcriptome profiles with clinically relevant *in vivo* models have identified IL-22-targeting as a prospective intervention in psoriasis (242) Guselkumab (anti-IL-23) acted as a long-time effector by suppressing IL-17 and IL-22 after withdrawal in the treatment of psoriasis, indicating that IL-22 blockers might be effective in both active and maintenance phases (231). Surprisingly, clinical trials of anti-IL-22 monoclonal antibodies (mAb), ILV-094 (NCT00563524) and ILV-095 (NCT01010542), are discontinued because of no efficacy in psoriasis patients with PASI ≥ 11 and PGA ≥ 3 in active phases (**Table 1**). The negative results of both trials might be due to that IL-22 acts on keratinocytes and induces abnormal proliferation and differentiation of the epidermis rather than inflammation. Notably, after years of treatment with both infliximab and ustekinumab, IL-22-expressing CD4+ T cells-maintained secretion of IL-22 after stimulation in clinically-healed-lesions (248). Thus, targeting IL-22 might be used as a maintenance treatment to avoid psoriasis relapse. In some cases, patients do not respond to common biologics. For example, Fania reported that three psoriasis patients developed psoriasiform skin lesions with augmented expression of IL-22 after receiving adalimumab (249). Megna also discovered increased levels of IL-22 in psoriasis patients who switched to eczematous drug eruption after treatment of anti-IL-17 biologics (250). Thus, combined therapies of anti-TNF-α (or anti-IL-23/IL-17) and anti-IL-22 intervention might be effective in anti-TNF-α (or anti-IL-23/IL-17)-resistant psoriasis individuals. Another reason for the failure of anti-IL-22 therapy in psoriasis might be the overlapping activities of IL-22, IL-20 and IL-24 (218). Thus, inhibiting IL-22 or IL-20 (251) alone might not be enough for a strong clinical response. Targeting IL-22R might have better clinical improvements than IL-22 neutralization since it also inhibits IL-20 and IL-24 (**Table 1**). Moreover, Michiels proposed a specific noncanonical STAT3 activation targeting by IL-22R1 Y-independent pathways, which not only relieved psoriatic skin but also protected IL-22-dependent barrier defense function at mucosal sites (252). However, lower serum IL-22 was detected in psoriasis patients with metabolic syndromes than that in patients without systemic complications (253). As IL-22 proved beneficial effects in metabolic syndromes (254, 255), anti-IL-22 treatment might deteriorate metabolic changes in psoriasis patients.

**Table 1:** Clinical Trials Targeted IL-22 Immune Response.

Intervention	Biologic Type	Year	Diseases	Status	Phase	SampleSize	Subjects	Primary Outcomes		Results	Trial	Reference
								Time Frame	Index			
**ILV-094** **(Fezakinumab)**	anti-IL-22 mAb IgG1	2007	PS	Completed	1	76	≥ 18 y	126 d	AEs	Not open	NCT00563524	–
								56-84 d	PASI, TLS, PGA	
		2007	HeS	Completed	1	56	20 y – 40 y	1 y	AEs, PK, PD	Not open	NCT00434746	–
		2007	HeS	Completed	1	56	18 y – 50 y	–	AEs, PK, PD	Not open	NCT00447681	–
		2009	RA	Completed	2	195	23 y – 81 y	12 w	ACR20	No efficacy	NCT00883896/ 2008-006936-37	–
		2013	AD	Completed	2	60	18 y – 75 y	12 w	SCORAD	Improve SCORAD	NCT01941537	(243), (244)
**ILV-095**	anti-IL-22 antagonist	2009	HeS	Completed	1	48	18 y – 50 y	3 w	AEs	Not open	NCT00822835	–
		2009	HeS	Completed	1	48	20 y – 45 y	3 w	AEs	Not open	NCT00822484	–
		2009	PS	Terminated	1	39	≥ 18 y	56 d	PASI, TLS, PGA	No efficacy	NCT01010542	–
**LEO 138559** **(ARGX-112 or LP 0145)**	IL-22R antagonists	2018	AD	Completed	1	47	18 y – 55 y	120 d	AEs	Not open	NCT03514511	–
		2021	AD	Recruiting	2	52	18 y – 64 y	16 w	EASI	–	NCT04922021	–
**F-652**	recombinant human IL-22 Fc IgG2	2012	HeS	Completed	1	40	18 y – 45 y	22 d	AEs	Safe & tolerable	ACTRN12612000713897	(245)
		2015	lower GI aGVHD	Completed	1, 2	27	18 y – 80 y	28 d	Response Rate	70.4%	NCT02406651	–
		2016	AH	Completed	1, 2	18	≥ 21 y	42 d	SAEs	Improve MELD scores	NCT02655510	(246)
		2020	COVID-19	Recruiting	2	38	≥ 18 y	29 d	NIAID	–	NCT04498377	–
**UTTR1147A** **(Efmarodocokin alfa or UTTR1147A or RG7880 or RO7021610)**	recombinant IL-22Fc IgG4	2014	HeS	Completed	1	68	18 y – 50 y	57 d	AEs, PK, PD	Safe & tolerable	2014-002252-10	(247)
		2016	NDFU	Completed	1	72	≥ 18 y	141 d	AEs	Not open	NCT02833389	–
		2016	UC, CD	Completed	1	70	18 y – 80 y	134 d	AEs	Not open	NCT02749630	–
		2018	UC, CD	Recruiting	2	320	18 y – 80 y	2 y	AEs	–	NCT03650413	–
		2018	UC	Not Recruiting	2	195	18 y – 80 y	8 w	Clinical Remission	–	NCT03558152	–
		2020	aGVHD	Recruiting	1	24	≥ 18 y	365 d	AEs	–	NCT04539470	–
		2020	COVID-19	Completed	2	410	≥ 18 y	28 d	Time to Recovery	Not open	NCT04386616	–

Data obtained from https://www.clinicaltrials.gov/, https://www.anzctr.org.au/, and https://www.clinicaltrialsregister.eu/ with search terms "ILV-094", ''ILV-095", "fezakinumab", "F-652", "Efmarodocokin alfa", "UTTR1147A", "RG-7880", "RG7880", "RO 7021610", “LEO 138559”, “ARGX-112”, “LP 0145” (Deadline: September 26, 2021).

mAb, monoclonal antibody; PS, Psoriasis; HeS, Healthy Subjects; AD, Atopic Dermatitis; AA, Alopecia Areata; SLE, Systemic Lupus Erythematosus; aGVHD, acute Graft-Versus-Host-Disease; AH, Alcoholic Hepatitis; NDFU, Neuropathic Diabetic Foot Ulcers; UC, Ulcerative Colitis; CD, Crohn's Disease; DMARD, Disease-modifying antirheumatic drugs; NIAID, the National Institute of Allergy and Infectious Diseases; SCORAD, the Scoring of Atopic Dermatitis; PASI, Psoriasis Area and Severity Index; TLS, Target Lesion Score; PGA, Physician global assessment; ACR20, American College of Rheumatology 20% improvement; EASI, Eczema Area and Severity Index; AEs, Adverse Events; SAEs, Serious Adverse Events; PK, Pharmacokinetic; PD, Pharmacodynamics; MELD, Model for End-Stage Liver Disease; d, day; w, week; y, year.

Different from the negative results of anti-IL-22 in psoriasis trials, ILV-094 seems attractive in AD therapeutics, as it improved SCORAD and neutrophils infiltration of asthma in severe AD patients in a preliminary study (243, 244) (**Table 1**). Dupilumab could inhibit IL-4, IL-13 and IL-22 simultaneously (256). However, some refractory AD patients respond poorly to dupilumab. Interestingly, a mathematical model revealed that simultaneous inhibition of IL-13 and IL-22 would be a promising intervention for them (257). Conversely, recombinant IL-22, F-652 or UTTR1147A, might strengthen its protective role in IL-22-deficient diseases such as GVHD and ulcer (**Table 1**).

In summary, Th22 cells represent a novel Th cell subset. However, their differentiation and regulation mechanisms remain to be fully elucidated. Emerging evidences have suggested that Th22 cells and IL-22 can play either protective or pathogenetic roles in various skin disorders. The biological roles of IL-22 depend on the local environment and disease setting contexts. Further studies are required to elucidate the mechanisms of Th22 cells in the pathogenesis of different diseases, as well as explore therapeutics targeting the Th22/IL-22 signaling pathway in skin disorders. Non-invasive transdermal delivery systems, such as microneedles with cell-penetrating peptides (258), might expand our application of biologics in the future.

## Author Contributions

YP drafted and edited the manuscript, DD, LW and XW drafted and edited the figure and tables, GH and XJ revised the manuscript. All authors contributed to the article and approved the submitted version.

## Funding

This work was supported by the National Natural Science Foundation of China (81872535, 22177084 and 82073473), the Fundamental Research Funds of Science & Technology Department of Sichuan Province (Grant Nos. 2022YFQ0054) and the 1.3.5 project for disciplines of excellence, West China Hospital, Sichuan University (ZYJC21036).

## Conflict of Interest

The authors declare that the research was conducted in the absence of any commercial or financial relationships that could be construed as a potential conflict of interest.

## Publisher’s Note

All claims expressed in this article are solely those of the authors and do not necessarily represent those of their affiliated organizations, or those of the publisher, the editors and the reviewers. Any product that may be evaluated in this article, or claim that may be made by its manufacturer, is not guaranteed or endorsed by the publisher.
