# Supplementary material for: The Role of T Helper 22 Cells in Dermatological Disorders
Source: Front Immunol. 2022 Jul 14;13:911546. doi: 10.3389/fimmu.2022.911546 (PMC9331286; doi:10.3389/fimmu.2022.911546)
Supplement: Supplementary file 1 [file Table_1.docx]

**Table S1 Cellular sources of IL-22**

| Cell type | Specific surface markers | Other cytokines expressed | Specific transcription factors | Inducers promoting IL-22 | Transcription factors promoting IL-22 | Reference |
| --- | --- | --- | --- | --- | --- | --- |
| NK cells | CD3-CD56+CD16+CD117-NKp46+ | INF-γ, TNF-α, GM-CSF | T-bet | IL-12, IL-2, IL-18 | STAT4 | (Duhen et al., 2009; Sabat et al., 2014; Dudakov et al., 2015) |
| ILC3 cells | Lin-CD117+ | GM-CSF | RORγt, T-bet, AHR | IL-23 | STAT3, T-bet, Notch | (Sabat et al., 2014; Dudakov et al., 2015; Bruggen et al., 2016; Bielecki et al., 2021) |
| LTi cells | C-kit+CCR6+ | IL-17, IL-22, lymphotoxin | RORγt, AHR | IL-23 | STAT3 | (Sabat et al., 2014; Arshad et al., 2020; Lindahl and Olsson, 2021) |
| NK T cells | CD3- | IFN-γ, IL-17 | - | IL-23, IL-1β | STAT3, RORγt, AHR | (Dudakov et al., 2015; Lindahl and Olsson, 2021) |
| γ𝛿T cells | CD3+γ𝛿TCR+ | IGF1, IL-17, IL-21 | - | IL-23 | STAT3, RORγt, AHR | (Sabat et al., 2014; Dudakov et al., 2015) (Malhotra et al., 2016) |
| Th1 cells | CD3+CD4+CXCR3+ | IFN-γ, IL-2, TNF-α/β | T-bet | IL-12, IL-18, IL-23 | STAT4 | (Sabat et al., 2014; Sabat et al., 2019) |
| Th17 cells | CD3+CD4+CCR4+CCR6+ | IL-17, IL-21, IL-26 | RORγt | IL-6, IL-23, IL-21, IL-1β, TGF-β | STAT3, RORγt, Notch, AHR | (Sabat et al., 2014; Dudakov et al., 2015; Sabat et al., 2019) |
| Th22 cells | CD3+CD4+CCR4+CCR6+CCR10+ | TNF-α | AHR | IL-6, TNF-α, IL-23, IL-1β | STAT3, AHR, Notch, RUNX1/3, HIF-1α | (Sabat et al., 2014; Sabat et al., 2019) |
| Tc17 cells | CD3+CD8+CCR4+CCR6+ | IFN-γ, IL-17 | RORγt | Unknown | STAT3 | (Sabat et al., 2019) |
| Tc22 cells | CD3+CD8+CCR4+CCR6+CCR10+ | TNF-α | AHR | IL-23, IL-21 | STAT1/3/5 | (Sabat et al., 2019; Arshad et al., 2020) |
| DC | CD11c+ | IL-12, IL-6, IL-23, TNF-α, IFN | - | IL-23, PRRs | STAT3, RORγt | (Zheng et al., 2008; Fumagalli et al., 2016; Nutt and Chopin, 2020) |
| Macrophage | CD11b+CD68+ | IL-17, IFN-γ | - | IL-23 | STAT3, RORγt | (Res et al., 2010; Hou et al., 2018) |
| Mast cell | CD3-c-Kit+FcεRI+ | IL-17 | - | - | - | (Mashiko et al., 2015; Ueshima et al., 2015) |
| Neutrophil | - | IL-17 | - | - | - | (Dyring-Andersen et al., 2017) |
| Cancer-associated fibroblasts | - | - | - | - | - | (Fukui et al., 2014; Li et al., 2019) |
| Immunofibroblasts | - | - | - | - | - | (Nayar et al., 2019) |

ILC, innate lymphoid cell; NKT, natural killer T cell; LTi, Lymphoid tissue inducer; 𝛄𝛿T, gamma-delta T cell; DC, dendritic cell; Th, T helper cell; AHR, aryl hydrocarbon receptor; GM-CSF, granulocyte-macrophage colony-stimulating factor; IGF1, insulin-like growth factor 1.

**Reference**

Arshad, T., Mansur, F., Palek, R., Manzoor, S., and Liska, V. (2020). A Double Edged Sword Role of Interleukin-22 in Wound Healing and Tissue Regeneration. *Front Immunol* 11**,** 2148. doi: 10.3389/fimmu.2020.02148.

Bielecki, P., Riesenfeld, S.J., Hütter, J.C., Torlai Triglia, E., Kowalczyk, M.S., Ricardo-Gonzalez, R.R., et al. (2021). Skin-resident innate lymphoid cells converge on a pathogenic effector state. *Nature* 592(7852)**,** 128-132. doi: 10.1038/s41586-021-03188-w.

Bruggen, M.C., Bauer, W.M., Reininger, B., Clim, E., Captarencu, C., Steiner, G.E., et al. (2016). In Situ Mapping of Innate Lymphoid Cells in Human Skin: Evidence for Remarkable Differences between Normal and Inflamed Skin. *J Invest Dermatol* 136(12)**,** 2396-2405. doi: 10.1016/j.jid.2016.07.017.

Dudakov, J.A., Hanash, A.M., and van den Brink, M.R. (2015). Interleukin-22: immunobiology and pathology. *Annu Rev Immunol* 33**,** 747-785. doi: 10.1146/annurev-immunol-032414-112123.

Duhen, T., Geiger, R., Jarrossay, D., Lanzavecchia, A., and Sallusto, F. (2009). Production of interleukin 22 but not interleukin 17 by a subset of human skin-homing memory T cells. *Nat Immunol* 10(8)**,** 857-863. doi: 10.1038/ni.1767.

Dyring-Andersen, B., Honoré, T.V., Madelung, A., Bzorek, M., Simonsen, S., Clemmensen, S.N., et al. (2017). Interleukin (IL)-17A and IL-22-producing neutrophils in psoriatic skin. *Br J Dermatol* 177(6)**,** e321-e322. doi: 10.1111/bjd.15533.

Fukui, H., Zhang, X., Sun, C., Hara, K., Kikuchi, S., Yamasaki, T., et al. (2014). IL-22 produced by cancer-associated fibroblasts promotes gastric cancer cell invasion via STAT3 and ERK signaling. *Br J Cancer* 111(4)**,** 763-771. doi: 10.1038/bjc.2014.336.

Fumagalli, S., Torri, A., Papagna, A., Citterio, S., Mainoldi, F., and Foti, M. (2016). IL-22 is rapidly induced by Pathogen Recognition Receptors Stimulation in Bone-Marrow-derived Dendritic Cells in the Absence of IL-23. *Sci Rep* 6**,** 33900. doi: 10.1038/srep33900.

Hou, Y., Zhu, L., Tian, H., Sun, H.X., Wang, R., Zhang, L., et al. (2018). IL-23-induced macrophage polarization and its pathological roles in mice with imiquimod-induced psoriasis. *Protein Cell* 9(12)**,** 1027-1038. doi: 10.1007/s13238-018-0505-z.

Li, H., Zhang, Q., Wu, Q., Cui, Y., Zhu, H., Fang, M., et al. (2019). Interleukin-22 secreted by cancer-associated fibroblasts regulates the proliferation and metastasis of lung cancer cells via the PI3K-Akt-mTOR signaling pathway. *Am J Transl Res* 11(7)**,** 4077-4088.

Lindahl, H., and Olsson, T. (2021). Interleukin-22 Influences the Th1/Th17 Axis. *Front Immunol* 12**,** 618110. doi: 10.3389/fimmu.2021.618110.

Malhotra, N., Yoon, J., Leyva-Castillo, J.M., Galand, C., Archer, N., Miller, L.S., et al. (2016). IL-22 derived from γδ T cells restricts Staphylococcus aureus infection of mechanically injured skin. *J Allergy Clin Immunol* 138(4)**,** 1098-1107.e1093. doi: 10.1016/j.jaci.2016.07.001.

Mashiko, S., Bouguermouh, S., Rubio, M., Baba, N., Bissonnette, R., and Sarfati, M. (2015). Human mast cells are major IL-22 producers in patients with psoriasis and atopic dermatitis. *J Allergy Clin Immunol* 136(2)**,** 351-359.e351. doi: 10.1016/j.jaci.2015.01.033.

Nayar, S., Campos, J., Smith, C.G., Iannizzotto, V., Gardner, D.H., Mourcin, F., et al. (2019). Immunofibroblasts are pivotal drivers of tertiary lymphoid structure formation and local pathology. *Proc Natl Acad Sci U S A* 116(27)**,** 13490-13497. doi: 10.1073/pnas.1905301116.

Nutt, S.L., and Chopin, M. (2020). Transcriptional Networks Driving Dendritic Cell Differentiation and Function. *Immunity* 52(6)**,** 942-956. doi: 10.1016/j.immuni.2020.05.005.

Res, P.C., Piskin, G., de Boer, O.J., van der Loos, C.M., Teeling, P., Bos, J.D., et al. (2010). Overrepresentation of IL-17A and IL-22 producing CD8 T cells in lesional skin suggests their involvement in the pathogenesis of psoriasis. *PLoS One* 5(11)**,** e14108. doi: 10.1371/journal.pone.0014108.

Sabat, R., Ouyang, W., and Wolk, K. (2014). Therapeutic opportunities of the IL-22-IL-22R1 system. *Nat Rev Drug Discov* 13(1)**,** 21-38. doi: 10.1038/nrd4176.

Sabat, R., Wolk, K., Loyal, L., Docke, W.D., and Ghoreschi, K. (2019). T cell pathology in skin inflammation. *Semin Immunopathol* 41(3)**,** 359-377. doi: 10.1007/s00281-019-00742-7.

Ueshima, C., Kataoka, T.R., Hirata, M., Koyanagi, I., Honda, T., Tsuruyama, T., et al. (2015). NKp46 regulates the production of serine proteases and IL-22 in human mast cells in urticaria pigmentosa. *Exp Dermatol* 24(9)**,** 675-679. doi: 10.1111/exd.12741.

Zheng, Y., Valdez, P.A., Danilenko, D.M., Hu, Y., Sa, S.M., Gong, Q., et al. (2008). Interleukin-22 mediates early host defense against attaching and effacing bacterial pathogens. *Nat Med* 14(3)**,** 282-289. doi: 10.1038/nm1720.

**Table S2 Clinical Trials Related with Th22/IL-22 in Skin Diseases**

| Diseases | First Posted | Sample Size | Intervention | Study Title | Th22/IL-22 Related Outcomes | Status | Study Phase | Trial | Publication |
| --- | --- | --- | --- | --- | --- | --- | --- | --- | --- |
| Psoriasis | 2007 | 31 | Apremilast | Apremilast Safety and PK Study in Recalcitrant Plaque Psoriasis | L-22 (skin) | Completed | 2 | NCT00521339 | Apremilast decreased IL-22 at week 12(Gottlieb et al., 2013) |
|  | 2009 | 39 | ILV-095 | Study Evaluating Single Dose Of ILV-095 In Psoriasis Subjects | IL-22 | Terminated | 1 | NCT01010542 | - |
|  | 2010 | 130 | NA | Improving Psoriasis Through Health and Well-Being | IL-22 (blood) | Completed | NA | NCT01162252 | - |
|  | 2011 | 40 | Ustekinumab, Etanercept | A Study to Assess the Effect of Ustekinumab (Stelara®) and Etanercept (Enbrel®) in Participants With Moderate to Severe Psoriasis (MK-0000-206) | IL-22 (skin) | Completed | 1 | NCT01276847 | - |
|  | 2014 | 20 | Indigo naturalis extract in oil ointment | Mechanistic Study of Indigo Naturalis in Treating Psoriasis | IL-22 (blood) | Completed | 2 | NCT02088281 | - |
|  | 2014 | 16 | Ustekinumab (Stelara) | Mechanism of action study of Ustekinumab treatment in psoriatic arthritis: Impact on cellular and molecular pathways of synovial inflammation and tissue remodeling | IL-22 (synovial) | Ongoing | 4 | EudraCT: 2014-003148-11 | - |
|  | 2016 | 65 | GSK2982772 | Safety, Tolerability, Pharmacokinetics, Pharmacodynamics, and Efficacy of Repeat Doses of GSK2982772 in Subjects With Psoriasis | IL-22 (skin, blood) | Completed | 2 | NCT02776033 | No difference of IL-22 mRNA observed(Weisel et al., 2020) |
|  | 2018 | 9 | NA | Study of Molecular Markers in Cutaneous Inflammation Between Psoriatic Lesional Skin and Healthy Non-lesional Skin (EMIP) | miR-21, IL-22 (skin) | Completed | NA | NCT03423004 | - |
|  | 2018 | 100 | NA | Identification of New Prognostic Markers in Psoriatic Arthritis | IL-22 (blood) | Completed | NA | NCT03455166 | Not mention IL-22 (Diani et al., 2019) |
|  | 2019 | 880 | Guselkumab | A Study to Evaluate Further Therapeutic Strategies With Guselkumab in Participants With Moderate-to-Severe Plaque-Type Psoriasis (GUIDE) | IL-22 (blood) | Active, not recruiting | 3 | NCT03818035,  EudraCT: 2018-001238-16 | - |
|  | 2019 | 156 | IMU-935 | A three-part, double-blind, placebo-controlled, phase I/Ib study of the safety, tolerability and pharmacokinetics of single and multiple ascending doses of IMU-935 in healthy volunteers and Psoriasis Patients. | IL-22 (blood) | recruiting | 1 | ACTRN12619001544167 | - |
|  | 2019 | 24 | FPP003 | A Phase 1/2a Study to Assess the Safety, Tolerability, Pharmacokinetics, Pharmacodynamic Effects and Exploratory Efficacy of FPP003 Vaccine in Subjects with Psoriasis. | IL-22 (skin, blood) | Recruiting | 1, 2 | ACTRN12619000599178 | - |
|  | 2019 | 50 | Guselkumab (Tremfya) | An exploratory, single-center, double-blinded, healthy volunteer controlled study to characterize psoriasis patients and explore novel biomarkers for the treatment response of psoriasis with a multi-modal patient profiling approach | IL-22 (blood, blister fluid) | Ongoing | 4 | EudraCT: 2019-002383-27 | - |
|  | 2019 | 10 | Secukinumab (Cosentyx) | investigation of inflammatory tendon Insertion by biopsy | IL-22 (enthesis) | Ongoing | 4 | EudraCT: 2018-004734-15 | - |
|  | 2020 | 270 | Glucocorticoid/DMARD | Impact of Tapering Immunosuppressants on Maintaining Minimal Disease Activity in Adult Subjects With Psoriatic Arthritis | IL-22 (blood) | Not yet recruiting | 3 | NCT04610476 | - |
|  | 2021 | 294 | Ustekinumab (AVT04, US Stelara, EU Stelara) | Pharmacokinetics, Safety and Tolerability Study of AVT04 to EU Approved and US Licensed Stelara (Ustekinumab) | IL-22 (blood) | Recruiting | 1 | NCT04744363 | - |
| Atopic Dermatitis | 2012 | 9 | NA | Immune Reactions in Contact Dermatitis Affected Skin | IL-22/th-22 (skin) | Completed | NA | NCT01546298 | - |
|  | 2013 | 60 | ILV-094 | A Randomized Placebo-controlled Study to Determine the Safety, Tolerability, Pharmacodynamics and Clinical Efficacy of ILV-094 (an IL-22 Antibody) Administered Intravenously to Subjects With Atopic Dermatitis (AD) | IL-22 (skin) | Completed | 2 | NCT01941537 | ILV-094 (anti-IL-22) improved clinical and molecular disease scores at week 12 with sustained responses and favorable safeties at week 20  (Guttman-Yassky et al., 2018),  ILV-094-response scores correlated with TH22/IL-22 pathways, but not with IL-22 mRNA across tissue in AD with asthma.(Badi et al., 2021) |
|  | 2014 | 45 | Whey proteins | Effectiveness of whey proteins for the treatment of atopic dermatitis: A pilot study. | IL-22 (blood) | Completed | 1, 2 | ACTRN12614000635662 | Not mention IL-22 (Tong et al., 2017) |
|  | 2016 | 34 | Jaungo | Efficacy, Safety and Dose Finding Trial of Topical Jaungo Application in Atopic Dermatitis Patients | IL-22 | Completed | 2 | NCT02900131 | NA (protocol)(Yun et al., 2017) |
|  | 2017 | 40 | Crisaborole | Crisaborole Ointment 2% Skin Biomarker Biopsy Study in Atopic Dermatitis | IL-22 | Completed | 2 | NCT03233529 | Crisaborole decreased Th1/Th22 pathway biomarkers (S100A7/8/9/12, CXCL1/2, PI3/elafin,  and LCN2) at day 8 and day 15(Bissonnette et al., 2019) |
|  | 2018 | 16 | Narrow-band UVB | Atopic Dermatitis: Early Gene Expression Changes as Predictors of Therapeutic Response to Narrow-band UVB Treatment | Th22 Gene Expression Changes | Active, not recruiting | NA | NCT03402412 | - |
|  | 2019 | 51 | PF-04965842 | Study Evaluating the Mechanism of Action of PF-04965842 Monotherapy for Moderate-to-severe Atopic Dermatitis (JADE MOA) | S100A8, S100A9, S100A12 (skin) | recruiting | 2 | NCT03915496 | - |
|  | 2019 | 20 | Dupilumab | The Effects of Dupilumab on Allergic Contact Dermatitis | Th22 Immune Pathways (skin, blood) | recruiting | 4 | NCT03935971 | - |
|  | 2019 | 36 | Betamethasone (Betnovate), Tacrolimus (Protopic) | The effects of topical corticosteroid use on insulin sensitivity and bone turnover | IL-22 (skin) | Completed | 4 | EudraCT: 2018-004370-96 | - |
|  | 2019 | 20 | Dupilumab, Betneval, Dermoval | Dupilumab impact on skin resident memory T cells | IL-22 (skin, blood) | Ongoing | 3 | EudraCT: 2018-004073-27 | - |
|  | 2019 | 8 | Dupilumab (Dupixent) | An exploratory study to investigate the extent to which Dupilumab becomes available to the body as well as Its actions and effects in the liquid found between the skin cells of atopic dermatitis patients | IL-22 (blood, blister fluid) | Ongoing | 4 | EudraCT: 2018-003642-17 | - |
|  | 2020 | 45 | NA | The Role of IL-22/IL-22BP Axis in Atopic Dermatitis (DA/IL-22BP) | IL-22, IL-22BP, IL-22R, IL-22-induced genes (skin, blood) | Recruiting | NA | NCT04310189 | - |
|  | 2021 | 20 | NA | Staphylococcus Aureus in Atopic Dermatitis Immunopathology (STADE) | IL-22 (skin) | Not yet recruiting | NA | NCT04715087 | - |
|  | 2021 | 96 | FURESTEM-AD inj. (stem cell product) | Safety and Explore the Efficacy of Multiple Doses of FURESTEM-AD Inj. for Moderate to Severe Chronic Atopic Dermatitis | IL-22 | Enrolling by invitation | 1, 2 | NCT04725136 | - |
| AA | 2016 | 22 | Tralokinumab | A Pilot Study of Tralokinumab in Subjects With Moderate to Severe Alopecia Areata | "T22"/IL-22 (skin) | Completed | 2 | NCT02684097 | - |
| Renal Transplant Recipients | 2017 | 84 | UAB30 | A Biomarker Evaluation Trial of UAB30 in Renal Transplant Recipients at High Risk for Non-melanoma Skin Cancer | IL-22 (skin) | Recruiting | 1, 2 | NCT03327064 | - |
| Eczema | 2019 | 60 | Aspirin | PGE2/IL-22 Pathway in Various Forms of Eczema | IL-22 (skin, blood) | Not yet recruiting | NA | NCT04133506 | - |
| SLE | 2020 | 60 | Itolizumab [Bmab 600] (EQ001) | Study of EQ001 (Itolizumab) in Systemic Lupus Erythematosus With or Without Active Proliferative Nephritis (EQUALISE) | IL-22 | recruiting | 1 | NCT04128579 | - |
| Vitiligo | 2020 | 44 | Methotrexate | Combination of Methotrexate and Phototherapy Versus Phototherapy in Adults With Progressive Vitiligo (METVI) | IL-22 (skin) | Not yet recruiting | 2 | NCT04237103 | - |
|  | 2021 | 48 | Baricitinib | Evaluation of Effect and Tolerance of the Association of Baricitinib and Phototherapy Versus Phototherapy in Adults with Progressive Vitiligo (BARVIT) | IL-22 (blood) | Not yet recruiting | 2 | NCT04822584 | - |
| Melanoma | 2021 | 50 | Anti-PD-1 monoclonal antibody | Evaluation of Cytokine Biomarkers in Melanoma Patients During Immunotherapy | IL-22 | Recruiting | 2 | NCT04928365 | - |

Data obtained from https://www.clinicaltrials.gov/, https://www.anzctr.org.au/, and https://www.clinicaltrialsregister.eu/ with search terms "IL-22", "Th22". .(Deadline: September 26, 2021).

NA, Not Applicable; AA Alopecia Areata; SLE, Systemic Lupus Erythematosus.

**Reference**

Badi, Y.E., Pavel, A.B., Pavlidis, S., Riley, J.H., Bates, S., Kermani, N.Z., et al. (2021). Mapping atopic dermatitis and anti-IL-22 response signatures to type 2-low severe neutrophilic asthma. *J Allergy Clin Immunol*. doi: 10.1016/j.jaci.2021.04.010.

Bissonnette, R., Pavel, A.B., Diaz, A., Werth, J.L., Zang, C., Vranic, I., et al. (2019). Crisaborole and atopic dermatitis skin biomarkers: An intrapatient randomized trial. *J Allergy Clin Immunol* 144(5)**,** 1274-1289. doi: 10.1016/j.jaci.2019.06.047.

Diani, M., Perego, S., Sansoni, V., Bertino, L., Gomarasca, M., Faraldi, M., et al. (2019). Differences in Osteoimmunological Biomarkers Predictive of Psoriatic Arthritis among a Large Italian Cohort of Psoriatic Patients. *Int J Mol Sci* 20(22). doi: 10.3390/ijms20225617.

Gottlieb, A.B., Matheson, R.T., Menter, A., Leonardi, C.L., Day, R.M., Hu, C., et al. (2013). Efficacy, tolerability, and pharmacodynamics of apremilast in recalcitrant plaque psoriasis: a phase II open-label study. *J Drugs Dermatol* 12(8)**,** 888-897.

Guttman-Yassky, E., Brunner, P.M., Neumann, A.U., Khattri, S., Pavel, A.B., Malik, K., et al. (2018). Efficacy and safety of fezakinumab (an IL-22 monoclonal antibody) in adults with moderate-to-severe atopic dermatitis inadequately controlled by conventional treatments: A randomized, double-blind, phase 2a trial. *J Am Acad Dermatol* 78(5)**,** 872-881 e876. doi: 10.1016/j.jaad.2018.01.016.

Tong, P.L., West, N.P., Cox, A.J., Gebski, V.J., Watts, A.M., Dodds, A., et al. (2017). Oral supplementation with bovine whey-derived Ig-rich fraction and lactoferrin improves SCORAD and DLQI in atopic dermatitis. *J Dermatol Sci* 85(2)**,** 143-146. doi: 10.1016/j.jdermsci.2016.11.009.

Weisel, K., Berger, S., Papp, K., Maari, C., Krueger, J.G., Scott, N., et al. (2020). Response to Inhibition of Receptor-Interacting Protein Kinase 1 (RIPK1) in Active Plaque Psoriasis: A Randomized Placebo-Controlled Study. *Clin Pharmacol Ther* 108(4)**,** 808-816. doi: 10.1002/cpt.1852.

Yun, Y., Ko, Y., Ahn, J.H., Jang, B.H., Kim, K., Ko, S.G., et al. (2017). Topical application of Jaungo in atopic dermatitis patients: study protocol for a randomized, controlled trial. *Trials* 18(1)**,** 176. doi: 10.1186/s13063-017-1920-9.

**Table S3 Drugs that potentially target Th22/IL-22 signaling pathway in skin diseases**

| Target | Drug | Alias | Biologic Type | Effects on Th22/IL-22 | Skin Diseases |
| --- | --- | --- | --- | --- | --- |
| CCR4 | Mogamulizumab | KW-0761, AMG-761 | mAb IgG1/κ | possibly inhibit recruitment of Th22 | CTCL, MF, Sézary Syndrome |
| CCL20 | GSK3050002 | - | mAb IgG1 | possibly inhibit recruitment of Th22 via decreasing CCR6 | PsA |
| IL-6 | Clazakizumab | BMS-945429, ALD518 | mAb IgG1 | possibly inhibit differentiation of Th22 | PS |
| IL-6R | Sarilumab | CNTO 136, SAR153191, REGN88 | mAb IgG1 |  | Morphea, SLE, CLE |
|  | Tocilizumab | Atlizumab | mAb IgG1 |  | SSc, BD, DM, Melanoma, SLE |
| TNF-𝛂 | Infliximab | - | mAb IgG1/κ | inhibit expression of IL-22(Olejniczak-Staruch et al., 2020), possibly inhibit differentiation of Th22 | PS, PsA, PV, PPP, BD, HS, PG, TEN, DM |
|  | Adalimumab | - | mAb IgG1 | inhibit production of Th22 and IL-22(Goldminz et al., 2015; Luan et al., 2015; Olejniczak-Staruch et al., 2020) | HS, PS, PsA, PPP, PG, Sarcoidosis |
|  | Golimumab | CNTO-148 | mAb IgG1/κ | possibly inhibit differentiation of Th22 | PsA, Sarcoidosis |
|  | Certolizumab pegol | CDP870, PHA-738144 | mAb IgG1 | possibly inhibit differentiation of Th22 | PS, PsA |
| TNF-𝛂R | Etanercept | - | IgG Fc fusion protein | inhibit expression of IL-22(Zaba et al., 2007; Caproni et al., 2009),  possibly inhibit differentiation of Th22; while some found no changes of IL-22 in PS at week 2 | PS, PsA, PPP, LP, Vitiligo, DLE, CU, HS, SJS/TEN |
| AHR | Tapinarof | GSK2894512, WBI 1001, Benvitimod | Agonist | increase the production of IL-22 invitro while inhibit IL-22 in PS mouse model(Smith et al., 2017) | PS, AD |
| IL-1 | Anakinra | - | Recombinant IL-1Ra | increase production of IL-22 in HS(Tzanetakou et al., 2016; Li et al., 2017) | amicrobial pustulosis, CADM, HS, SS, AOSD, AD, FCU, FCAS |
|  | Canakinumab | ACZ885 | mAb IgG1/κ | possibly inhibit differentiation of Th22 | SS, AOSD, PG, PAPA-like syndrome, urticarial vasculitis, BD |
|  | Rilonacept | IL-1 Trap | Soluble decoy | possibly inhibit differentiation of Th22 | FCAS, CCU, SS |
|  | Gevokizumab | XOMA 052 | mAb IgG2/κ | possibly inhibit differentiation of Th22 | GPP, Acne |
|  | RPH-104 | Goflikicept | Inhibitor | possibly inhibit differentiation of Th22 | SS |
| p19 of IL-23 | Tildrakizumab | MK-3222, SCH 900222 | mAb IgG1κ | possibly inhibit differentiation of Th22(Zhou et al., 2021) | PS, BP, vitiligo, PsA |
|  | Risankizumab | BI 655066, ABBV-066 | mAb IgG | possibly inhibit differentiation of Th22(Zhou et al., 2021) | PS, HS, PsA, PPP, AD, |
|  | Guselkumab | CNTO1959 | mAb IgG1λ | inhibit production of IL-22 even effective after withdrawal(Gordon et al., 2019; Sweet et al., 2021) | PRP, HS, PS, PsA, Scleroderma, PPP |
|  | mirikizumab | LY3074828 | mAb IgG4-variant | possibly inhibit differentiation of Th22 | PS |
| p40 of IL-12 & IL-23 | Ustekinumab | CNTO 1275, L04AC05, TT20 | mAb IgG1κ | inhibit production of IL-22(Feldmeyer et al., 2017; Philipp et al., 2020; Cesaroni et al., 2021) | PS, SLE, BP, AD, DM, PPP, BD, Ichthyosis |
| Jak1 | Filgotinib | - | Inhibitor | inhibit production of IL-22(Roblin et al., 2021) | CLE |
|  | Upadacitinib | ABT-494 | Inhibitor | possibly inhibit IL-22(Zhou et al., 2021) | AD, SLE, HS, PsA |
|  | Abrocitinib | PF-04965842 | Inhibitor | possibly inhibit biological roles of IL-22 | AD, PS |
|  | Itacitinib | INCB054329 | Inhibitor | possibly inhibit biological roles of IL-22 | PS |
|  | SHR0302 | - | Inhibitor | possibly inhibit biological roles of IL-22 | AD, PsA, Vitiligo |
|  | Solcitinib | GSK2586184 | Inhibitor | possibly inhibit biological roles of IL-22 | SLE, PS |
|  | INCB054707 | - | Inhibitor | possibly inhibit biological roles of IL-22 | HS, Vitiligo |
|  | ARQ-252 | - | Inhibitor | possibly inhibit biological roles of IL-22 | Vitiligo, Eczema |
| Jak1/2 | CTP-543 | deuruxolitinib | deuterated form of Ruxolitinib | possibly inhibit biological roles of IL-22 | AA |
|  | Baricitinib | LY3009104, INCB028050 | Inhibitor | possibly inhibit production of IL-22(Uchiyama et al., 2022) | AD, SLE, AA, PS |
|  | Ruxolitinib | INC424, INCB18424 | Inhibitor | Inhibit expression of IL-22R1(Abikhair Burgo et al., 2018) | Vitiligo, HS, AD, AA, PS, SCC |
| Jak1/3 | Ifidancitinib | ATI-502, A-301 | Inhibitor | possibly inhibit biological roles of IL-22 | AA, AD |
|  | ATI-1777 | - | Inhibitor | possibly inhibit biological roles of IL-22 | AD |
|  | ATI-501 | - | Inhibitor | possibly inhibit biological roles of IL-22 | AA |
| Jak1/2/3 | Tofacitinib | CP 690550 | Inhibitor | inhibit IL-22 induced changes of S100A7 and EGR1(Srivastava et al., 2018) | SLE, Scleroderma, AA, DM |
|  | Jaktinib Hydrochloride | - | Inhibitor | possibly inhibit biological roles of IL-22 | AD, AA |
|  | Oclacitinib | - | Inhibitor | possibly inhibit biological roles of IL-22 | CAD |
| Jak1/2/3, Tyk2 | Delgocitinib | LEO-124249, JTE-052 | Inhibitor | possibly inhibit biological roles of IL-22 | DLE, Eczema, UV-induced allergic skin reactions |
|  | Peficitinib | ASP015K, JNJ-54781532 | Inhibitor | possibly inhibit biological roles of IL-22 | PS |
| Jak1/2/3, Tyk2, SYK | Gusacitinib | ASN002, EN3351, WHO 10976 | Inhibitor | possibly inhibit biological roles of IL-22 | AD, BCC, Hand Eczema |
| Tyk2 | Deucravacitinib | BMS-986165, WHO 11342 | Inhibitor | possibly inhibit biological roles of IL-22 | PS, PsA |
|  | PF-06826647 | - | Inhibitor | possibly inhibit biological roles of IL-22 | PS, HS |
|  | BMS-986202 | - | Inhibitor | possibly inhibit biological roles of IL-22 | PS |
| Jak1/Tyk2 | Bepocitinib | PF-06700841 | Inhibitor | possibly inhibit biological roles of IL-22 | PS, AA, HS, Vitiligo |

Data obtained from Pubmed and https://www.clinicaltrials.gov/. (Deadline: September 26, 2021).

mAb, monoclonal antibody; PS, Psoriasis; AD, Atopic Dermatitis; AA, Alopecia Areata; SLE, Systemic Lupus Erythematosus; CLE, Cutaneous Lupus Erythematosus; DLE, Discoid Lupus Erythematosus; BD, Behcet’s Disease; DM, Dermatomyositis; PsA, Psoriatic Arthritis; PPP: Palmoplantar Pustulosis; PRP, Pityriasis Rubra Pilaris; LP, Lichen Planus; CU, Chronic Urticaria; FCU, Familial Cold Urticaria; CCU, Cold Contact Urticaria; BP, Bullous Pemphigus; PV, Pemphigus Vulgaris; HS, Hidradenitis Suppurativa; FICZ, 6-formylindolo [3,2 -b] carbazole; AHR, Aryl Hydrocarbon Receptor; CADM, Clinically Amyopathic Dermatomyopathy; CAD, Canine Allergic Dermatitis; AOSD, Adult Onset Still's Disease; FCAS, Familial Cold Autoinflammatory Syndrome; PG, Pyoderma Gangrenosum; SSc, Systemic Sclerosis; GPP, Generalized Pustular Psoriasis; TEN, Toxic Epidermal Necrolysis; SJS, Stevens-Johnson Syndrome; BCC, Basal Cell Carcinoma; SCC, Squamous Cell Carcinoma; CTCL, Cutaneous T-cell lymphoma; MF, Mycosis Fungoides; SS, Schnitzler's syndrome.

**Reference**

Badi, Y.E., Pavel, A.B., Pavlidis, S., Riley, J.H., Bates, S., Kermani, N.Z., et al. (2021). Mapping atopic dermatitis and anti-IL-22 response signatures to type 2-low severe neutrophilic asthma. *J Allergy Clin Immunol*. doi: 10.1016/j.jaci.2021.04.010.

Bissonnette, R., Pavel, A.B., Diaz, A., Werth, J.L., Zang, C., Vranic, I., et al. (2019). Crisaborole and atopic dermatitis skin biomarkers: An intrapatient randomized trial. *J Allergy Clin Immunol* 144(5)**,** 1274-1289. doi: 10.1016/j.jaci.2019.06.047.

Diani, M., Perego, S., Sansoni, V., Bertino, L., Gomarasca, M., Faraldi, M., et al. (2019). Differences in Osteoimmunological Biomarkers Predictive of Psoriatic Arthritis among a Large Italian Cohort of Psoriatic Patients. *Int J Mol Sci* 20(22). doi: 10.3390/ijms20225617.

Gottlieb, A.B., Matheson, R.T., Menter, A., Leonardi, C.L., Day, R.M., Hu, C., et al. (2013). Efficacy, tolerability, and pharmacodynamics of apremilast in recalcitrant plaque psoriasis: a phase II open-label study. *J Drugs Dermatol* 12(8)**,** 888-897.

Guttman-Yassky, E., Brunner, P.M., Neumann, A.U., Khattri, S., Pavel, A.B., Malik, K., et al. (2018). Efficacy and safety of fezakinumab (an IL-22 monoclonal antibody) in adults with moderate-to-severe atopic dermatitis inadequately controlled by conventional treatments: A randomized, double-blind, phase 2a trial. *J Am Acad Dermatol* 78(5)**,** 872-881 e876. doi: 10.1016/j.jaad.2018.01.016.

Tong, P.L., West, N.P., Cox, A.J., Gebski, V.J., Watts, A.M., Dodds, A., et al. (2017). Oral supplementation with bovine whey-derived Ig-rich fraction and lactoferrin improves SCORAD and DLQI in atopic dermatitis. *J Dermatol Sci* 85(2)**,** 143-146. doi: 10.1016/j.jdermsci.2016.11.009.

Weisel, K., Berger, S., Papp, K., Maari, C., Krueger, J.G., Scott, N., et al. (2020). Response to Inhibition of Receptor-Interacting Protein Kinase 1 (RIPK1) in Active Plaque Psoriasis: A Randomized Placebo-Controlled Study. *Clin Pharmacol Ther* 108(4)**,** 808-816. doi: 10.1002/cpt.1852.

Yun, Y., Ko, Y., Ahn, J.H., Jang, B.H., Kim, K., Ko, S.G., et al. (2017). Topical application of Jaungo in atopic dermatitis patients: study protocol for a randomized, controlled trial. *Trials* 18(1)**,** 176. doi: 10.1186/s13063-017-1920-9.

Abikhair Burgo, M., Roudiani, N., Chen, J., Santana, A.L., Doudican, N., Proby, C., et al. (2018). Ruxolitinib inhibits cyclosporine-induced proliferation of cutaneous squamous cell carcinoma. *JCI Insight* 3(17). doi: 10.1172/jci.insight.120750.

Caproni, M., Antiga, E., Melani, L., Volpi, W., Del Bianco, E., and Fabbri, P. (2009). Serum levels of IL-17 and IL-22 are reduced by etanercept, but not by acitretin, in patients with psoriasis: a randomized-controlled trial. *J Clin Immunol* 29(2)**,** 210-214. doi: 10.1007/s10875-008-9233-0.

Cesaroni, M., Seridi, L., Loza, M.J., Schreiter, J., Sweet, K., Franks, C., et al. (2021). Suppression of Serum Interferon-gamma Levels as a Potential Measure of Response to Ustekinumab Treatment in Patients With Systemic Lupus Erythematosus. *Arthritis Rheumatol* 73(3)**,** 472-477. doi: 10.1002/art.41547.

Feldmeyer, L., Mylonas, A., Demaria, O., Mennella, A., Yawalkar, N., Laffitte, E., et al. (2017). Interleukin 23-Helper T Cell 17 Axis as a Treatment Target for Pityriasis Rubra Pilaris. *JAMA Dermatol* 153(4)**,** 304-308. doi: 10.1001/jamadermatol.2016.5384.

Goldminz, A.M., Suárez-Fariñas, M., Wang, A.C., Dumont, N., Krueger, J.G., and Gottlieb, A.B. (2015). CCL20 and IL22 Messenger RNA Expression After Adalimumab vs Methotrexate Treatment of Psoriasis: A Randomized Clinical Trial. *JAMA Dermatol* 151(8)**,** 837-846. doi: 10.1001/jamadermatol.2015.0452.

Gordon, K.B., Armstrong, A.W., Foley, P., Song, M., Shen, Y.K., Li, S., et al. (2019). Guselkumab Efficacy after Withdrawal Is Associated with Suppression of Serum IL-23-Regulated IL-17 and IL-22 in Psoriasis: VOYAGE 2 Study. *J Invest Dermatol* 139(12)**,** 2437-2446.e2431. doi: 10.1016/j.jid.2019.05.016.

Li, J., Shouval, D.S., Doty, A.L., Snapper, S.B., and Glover, S.C. (2017). Increased Mucosal IL-22 Production of an IL-10RA Mutation Patient Following Anakin Treatment Suggests Further Mechanism for Mucosal Healing. *J Clin Immunol* 37(2)**,** 104-107. doi: 10.1007/s10875-016-0365-3.

Luan, L., Han, S., Wang, H., and Liu, X. (2015). Down-regulation of the Th1, Th17, and Th22 pathways due to anti-TNF-alpha treatment in psoriasis. *Int Immunopharmacol* 29(2)**,** 278-284. doi: 10.1016/j.intimp.2015.11.005.

Olejniczak-Staruch, I., Narbutt, J., Bednarski, I., Wozniacka, A., Sieniawska, J., Kraska-Gacka, M., et al. (2020). Interleukin 22 and 6 serum concentrations decrease under long-term biologic therapy in psoriasis. *Postepy Dermatol Alergol* 37(5)**,** 705-711. doi: 10.5114/ada.2020.100481.

Philipp, S., Menter, A., Nikkels, A.F., Barber, K., Landells, I., Eichenfield, L.F., et al. (2020). Ustekinumab for the treatment of moderate-to-severe plaque psoriasis in paediatric patients (>/= 6 to < 12 years of age): efficacy, safety, pharmacokinetic and biomarker results from the open-label CADMUS Jr study. *Br J Dermatol* 183(4)**,** 664-672. doi: 10.1111/bjd.19018.

Roblin, X., Serone, A., Yoon, O.K., Zhuo, L., Grant, E., Woo, J., et al. (2021). Effects of JAK1-Preferential Inhibitor Filgotinib on Circulating Biomarkers and Whole Blood Genes/Pathways of Patients With Moderately to Severely Active Crohn's Disease. *Inflamm Bowel Dis*. doi: 10.1093/ibd/izab253.

Smith, S.H., Jayawickreme, C., Rickard, D.J., Nicodeme, E., Bui, T., Simmons, C., et al. (2017). Tapinarof Is a Natural AhR Agonist that Resolves Skin Inflammation in Mice and Humans. *J Invest Dermatol* 137(10)**,** 2110-2119. doi: 10.1016/j.jid.2017.05.004.

Srivastava, A., Stahle, M., Pivarcsi, A., and Sonkoly, E. (2018). Tofacitinib Represses the Janus Kinase-Signal Transducer and Activators of Transcription Signalling Pathway in Keratinocytes. *Acta Derm Venereol* 98(8)**,** 772-775. doi: 10.2340/00015555-2960.

Sweet, K., Song, Q., Loza, M.J., McInnes, I.B., Ma, K., Leander, K., et al. (2021). Guselkumab induces robust reduction in acute phase proteins and type 17 effector cytokines in active psoriatic arthritis: results from phase 3 trials. *RMD Open* 7(2). doi: 10.1136/rmdopen-2021-001679.

Tzanetakou, V., Kanni, T., Giatrakou, S., Katoulis, A., Papadavid, E., Netea, M.G., et al. (2016). Safety and Efficacy of Anakinra in Severe Hidradenitis Suppurativa: A Randomized Clinical Trial. *JAMA Dermatol* 152(1)**,** 52-59. doi: 10.1001/jamadermatol.2015.3903.

Uchiyama, A., Fujiwara, C., Inoue, Y., Ishikawa, M., and Motegi, S.I. (2022). Possible suppressive effects of baricitinib on serum IL-22 levels in atopic dermatitis. *J Dermatol Sci*. doi: 10.1016/j.jdermsci.2022.04.006.

Zaba, L.C., Cardinale, I., Gilleaudeau, P., Sullivan-Whalen, M., Suárez-Fariñas, M., Fuentes-Duculan, J., et al. (2007). Amelioration of epidermal hyperplasia by TNF inhibition is associated with reduced Th17 responses. *J Exp Med* 204(13)**,** 3183-3194. doi: 10.1084/jem.20071094.

Zhou, L., Wang, Y., Wan, Q., Wu, F., Barbon, J., Dunstan, R., et al. (2021). A non-clinical comparative study of IL-23 antibodies in psoriasis. *MAbs* 13(1)**,** 1964420. doi: 10.1080/19420862.2021.1964420.
